# In Vitro Bioaccessibility of Bioactive Compounds from Citrus Pomaces and Orange Pomace Biscuits

**DOI:** 10.3390/molecules26123480

**Published:** 2021-06-08

**Authors:** Adriana Maite Fernández-Fernández, Eduardo Dellacassa, Tiziana Nardin, Roberto Larcher, Adriana Gámbaro, Alejandra Medrano-Fernandez, María Dolores del Castillo

**Affiliations:** 1 Departamento de Ciencia y Tecnología de Alimentos, Facultad de Química, Universidad de la República, General Flores 2124, Montevideo 11800, Uruguay; afernandez@fq.edu.uy (A.M.F.-F.); agambaro@fq.edu.uy (A.G.); amedrano@fq.edu.uy (A.M.-F.); 2 Instituto de Investigación en Ciencias de la Alimentación (CIAL) (CSIC-UAM), C/Nicolás Cabrera, 9, Campus de la Universidad Autónoma de Madrid, 28049 Madrid, Spain; 3 Graduate Program in Chemistry, Facultad de Química, Universidad de la República, General Flores 2124, Montevideo 11800, Uruguay; 4 Departamento de Química Orgánica, Facultad de Química, Universidad de la República, General Flores 2124, Montevideo 11800, Uruguay; edellac@fq.edu.uy; 5 Dipartimento Alimenti e Trasformazione, Centro Trasferimento Tecnologico, Fondazione Edmund Mach di San Michele all’Adige, Via E. Mach, 1 38010 Trento, Italy; tiziana.nardin@fmach.it (T.N.); roberto.larcher@fmach.it (R.L.)

**Keywords:** α-amylase, anti-inflammatory, antioxidant, bioaccessibility, citrus pomaces, diabetes, functional biscuits, α-glucosidase, sensory analysis

## Abstract

The present investigation aimed to provide novel information on the chemical composition and in vitro bioaccessibility of bioactive compounds from raw citrus pomaces (mandarin varieties Clemenule and Ortanique and orange varieties Navel and Valencia). The effects of the baking process on their bioaccessibility was also assessed. Samples of pomaces and biscuits containing them as an ingredient were digested, mimicking the human enzymatic oral gastrointestinal digestion process, and the composition of the digests were analyzed. UHPLC-MS/MS results of the citrus pomaces flavonoid composition showed nobiletin, hesperidin/neohesperidin, tangeretin, heptamethoxyflavone, tetramethylscutellarein, and naringin/narirutin. The analysis of the digests indicated the bioaccessibility of compounds possessing antioxidant [6.6–11.0 mg GAE/g digest, 65.5–97.1 µmol Trolox Equivalents (TE)/g digest, and 135.5–214.8 µmol TE/g digest for total phenol content (TPC), ABTS, and ORAC-FL methods, respectively; significant reduction (*p* < 0.05) in Reactive Oxygen Species (ROS) formation under tert-butyl hydroperoxide (1 mM)-induced conditions in IEC-6 and CCD-18Co cells when pre-treated with concentrations 5–25 µg/mL of the digests], anti-inflammatory [significant reduction (*p* < 0.05) in nitric oxide (NO) production in lipopolysaccharide (LPS)-induced RAW264.7 macrophages], and antidiabetic (IC_50_ 3.97–11.42 mg/mL and 58.04–105.68 mg/mL for α-glucosidase and α-amylase inhibition capacities) properties in the citrus pomaces under study. In addition, orange pomace biscuits with the nutrition claims “no-added sugars” and “source of fiber”, as well as those with good sensory quality (6.9–6.7, scale 1–9) and potential health promoting properties, were obtained. In conclusion, the results supported the feasibility of citrus pomace as a natural sustainable source of health-promoting compounds such as flavonoids. Unfractionated orange pomace may be employed as a functional food ingredient for reducing the risk of pathophysiological processes linked to oxidative stress, inflammation, and carbohydrate metabolism, such as diabetes, among others.

## 1. Introduction

During commercial juice production, large amounts of industrial waste are generated which are composed of pulp, seeds, and peels that account for about 50% of the total weight of citrus fruit. The use of this waste is fundamental for the fruit processing industry, not only for economic reasons, but also to reduce the severe environmental impact that it could produce [1]. Mandarins and oranges are a rich and sustainable source of flavonoids hesperetin and its disaccharide derivative (hesperidin), naringenin and its derivatives (narirutin and naringin), nobiletin, and dietary fiber, and thus have the potential for diabetes risk reduction or for the alleviation of its symptoms, such as hyperglycemia [1], which is considered a 21st century pandemic [2]. Chronic inflammation and oxidative stress are related to diabetes pathogenesis [3]. Some strategies (such as inhibition of carbohydrases and reduction in oxidant species) have been previously reported for diabetes control [4,5,6].

In order to have an effect on health, bioactive compounds have to withstand food processing and gastrointestinal tract conditions, be released from the food matrix in the gastrointestinal tract (bioaccessible) so they can be metabolized, and reach the target tissue of interest so they can exert their bioactive properties directly in the intestine. Polyphenols’ chemical structure may be transformed or form complexes with macromolecules in the food matrix during digestion, changing their bioactivities [7]. Thus, bioaccessibility studies are of critical importance in order to determine the remaining bioactive properties in citrus by-products. These bioactive compounds may be protected from degradation during digestion and food processing by the dietary fiber composing the by-product, as it is known that these phenolic compounds are mainly bound to cell wall structural components (bound phenolics) in fruits and vegetables, such as pectin [8,9]. In fact, thermal processing could enhance the release of bound phenolics by disrupting the cell wall of fruits and vegetables [9], with a consequent increase in their bioaccessibility [10].

The aim of this work was to provide the chemical composition and novel information regarding the in vitro bioaccessibility of the bioactive compounds present in raw citrus pomaces with the potential to reduce the risk of diabetes or to control it. In addition, biscuits with citrus by-products as food ingredients were obtained, along with their food sensory quality, and the in vitro bioactivity of biscuits’ bioaccessible fractions was evaluated in order to study the food matrix effect on their bioaccessibility.

## 2. Results and Discussion

### 2.1. Chemical Characterization of Citrus Pomaces by UHPLC-MS/MS Analysis

Nobiletin was the main flavonoid found in mandarin and orange pomaces (Table 1). Other flavonoids found in mandarin and orange pomaces, following nobiletin, were hesperidin/neohesperidin, tangeretin, heptamethoxyflavone, tetramethylscutellarein, and naringin/narirutin. The results are in agreement with previous reports [11,12,13,14,15]. Among mandarin pomace varieties, heptamethoxyflavone content was much higher (86%) for Clemenule mandarin pomace. On the other hand, naringin/narirutin content was much higher (71%) for Ortanique mandarin pomace. In the Supplementary Material, the plots of representative extracted ion chromatograms (EICs) for both positive and negative electrospray ionization are shown in Figure S1. Among orange pomace varieties, flavonoid profiles were similar. All these flavonoids have been found to possess numerous bioactive properties such as antioxidant, antidiabetic, and anti-inflammatory properties [1], representing great potential as functional ingredients.

### 2.2. Bioaccessibility of Antioxidants from Citrus Pomaces

**Figure 1:**
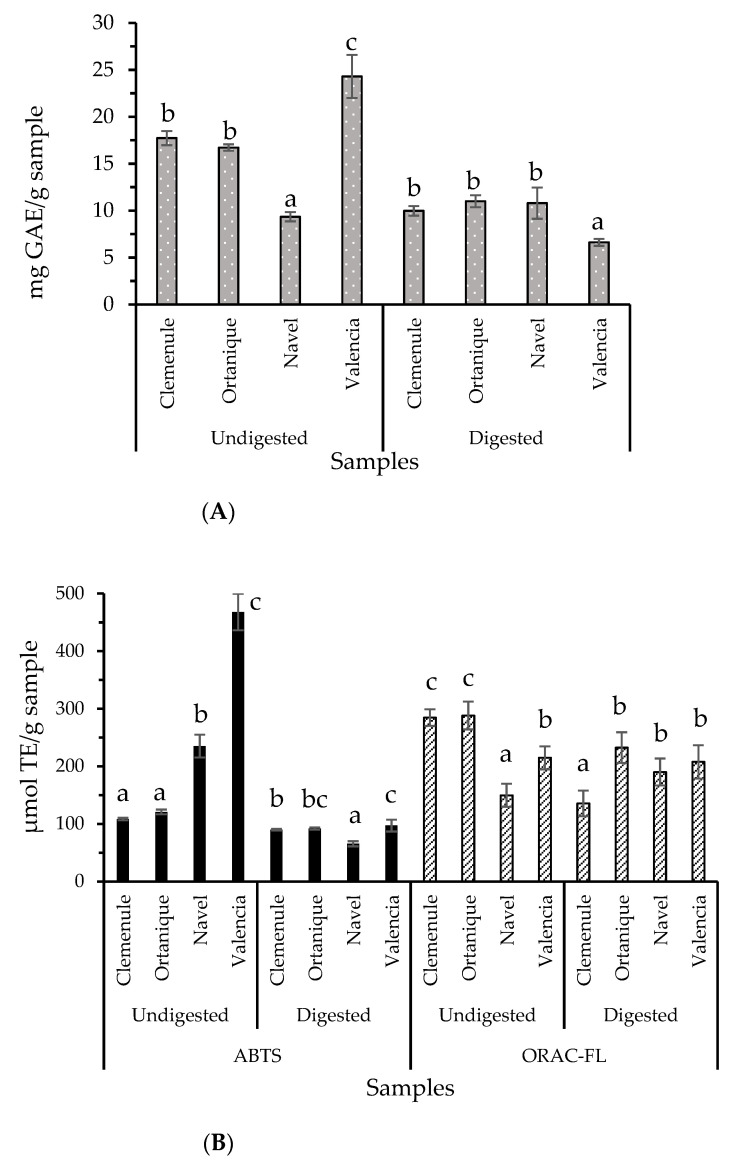
Antioxidants composing undigested and digested raw citrus pomaces: (**A**) Total Phenol Content (TPC). (**B**) ABTS and ORAC-FL assays. Bars and error bars represent mean values and standard deviation, respectively. Different letters (a, b and c) indicate significant differences (Tukey, *p* < 0.05) between values in each set of four samples where sets of samples are undigested ABTS samples, digested ABTS samples, undigested ORAC-FL samples, and digested ORAC-FL samples.

The presence of antioxidant compounds was confirmed by TPC, ABTS, and ORAC-FL methods, showing decreased presence after digestion in most of the samples and performed methods (Figure 1). Still, results showed remaining antioxidant compounds after the digestion process, which include polyphenolic compounds. Previous polyphenol bioaccessibility studies on citrus samples are in agreement with the current results [16]. Hesperidin, narirutin, naringenin-7-*O*-glucoside, and rutin have been found to remain in the bioaccessible fractions of Clementine mandarin and Navel orange pulps [16], which are present in the raw citrus pomaces of the current work.

Antioxidant compounds’ loss by means of the digestion process has already been reported for oregano samples [7], persimmon fruit byproduct flours [17], wild blueberries [18], and red grape skin [19]. Specifically, a study conducted on citrus peel extracts showed that flavonoids and antioxidant activity were sensitive to pH conditions during in vitro gastrointestinal digestion [20]. This loss may be the result of pH changes during the in vitro simulation of digestion in each phase, which led to a change in the polyphenols’ antioxidant capacity [7]. In most of the samples, antioxidant capacity analyzed by an ORAC-FL assay was unchanged or increased after digestion, suggesting the release of antioxidant compounds with the hydrogen atom transfer (HAT) mechanism of action during the digestion process. This bioactive compound release may be able to exert health benefits at the intestinal level.

**Figure 2:**
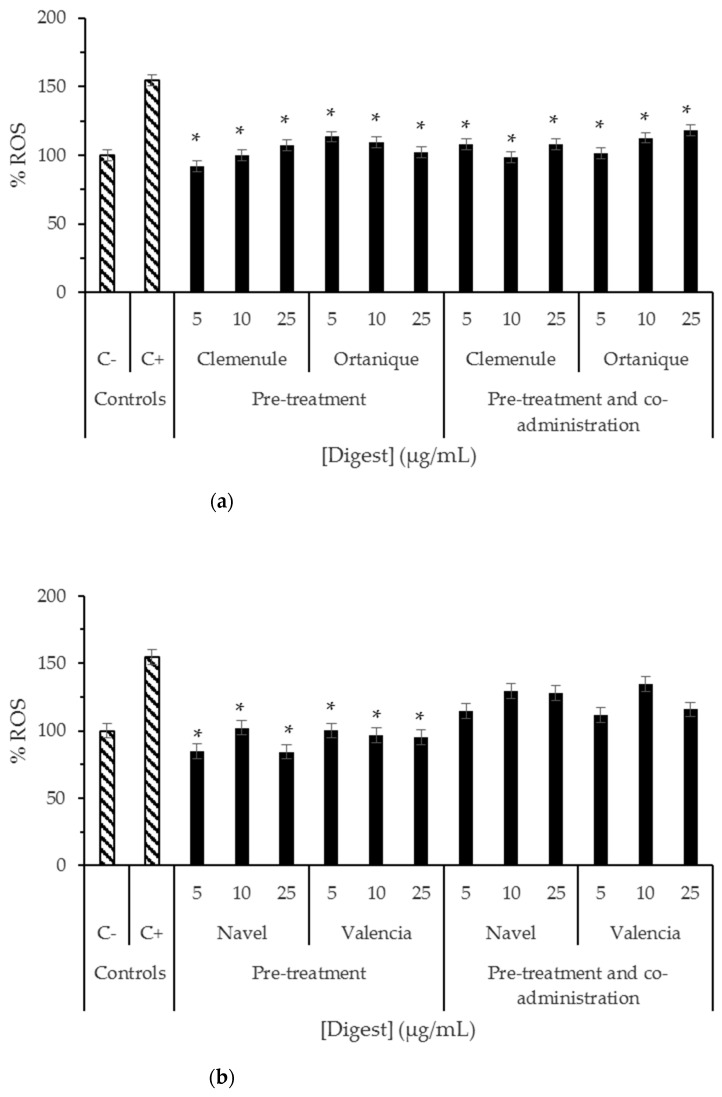
Induced ROS formation in normal small intestinal cells of rats (IEC-6) treated with digests of (**a**) Clemenule and Ortanique mandarin and (**b**) Navel and Valencia orange pomaces prior to oxidative damage. Cells treated with tert-butyl hydroperoxide were used as positive control (C+), while untreated cells were employed as negative control (C−). The basal production (100%, C-), was significantly increased due to the treatment with tert-butyl hydroperoxide until about 150% (C+). Pre-treatment assay: normal rat small intestinal cells were treated with samples for 24 h followed by administration of the oxidative agent (tert-butyl hydroperoxide) for 30 min. Pre-treatment and co-administration assay: cells were treated with samples for 24 h followed by co-administration of the oxidative agent (tert-butyl hydroperoxide) and sample for 30 min. Bars represent mean values while error bars denote standard error of the mean (SEM). * Indicates statistical differences between mean values with *p* < 0.05 by Tukey test when compared to the positive control (C+).

**Figure 3:**
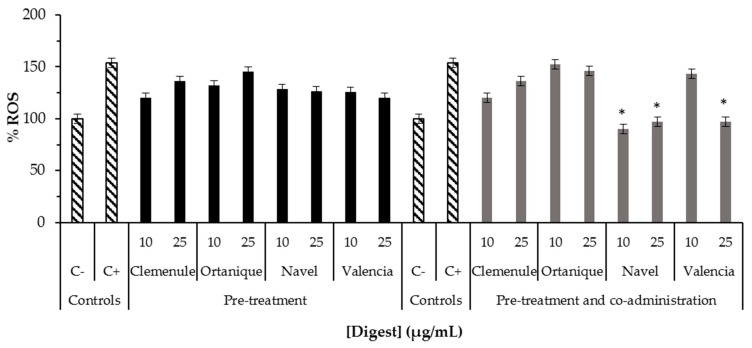
Induced ROS formation in normal human intestinal cells (CCD-18Co) treated with digests of Clemenule and Ortanique mandarin and Navel and Valencia orange pomaces prior to oxidative damage. Cells treated with tert-butyl hydroperoxide were used as positive control (C+), while untreated cells were employed as negative control (C−). The formation of ROS in the negative control, fixed as the 100% basal production, was significantly increased due to the treatment with tert-butyl hydroperoxide until about 150% (C+). Pre-treatment assay: normal human colon cells were treated with samples for 24 h followed by administration of the oxidative agent (tert-butyl hydroperoxide) for 30 min. Pre-treatment and co-administration assay: cells were treated with samples for 24 h followed by co-administration of the oxidative agent (tert-butyl hydroperoxide) and sample for 30 min. Bars represent mean values while error bars denote standard error of the mean (SEM). * Indicates statistical differences between mean values with *p* < 0.05 by Tukey test when compared to the positive control (C+).

The antioxidant effect of digested raw citrus pomaces was studied (Figure 2 and Figure 3), and it was found that intracellular ROS formation in the normal small intestinal cells of rats (IEC-6 cells) was significantly reduced (*p* < 0.05) by mandarin and orange pomaces (Figure 2). However, in normal human colon cells (CCD-18Co cells), digests of orange pomace samples were the only ones able to cause significant reduction (*p* < 0.05) in ROS formation when pre-treated with the samples (Figure 3). Intracellular ROS formation in mouse macrophages (RAW264.7 cells) was not significantly reduced (*p* > 0.05) by the samples under study (data not shown). The results suggest Navel and Valencia orange pomaces may play an important role counteracting oxidative stress negative effects in the colon cells that are exposed on a daily basis to high ROS levels by means of a prolonged residence time [21].

Similar results of citrus samples were found by others in HepG2 cells [22] regarding inhibition of intracellular ROS formation. In the latter study, sweet orange (*Citrus sinensis*) peel water extracts were found to decrease ROS levels when a pre-treatment for 24 h was performed with the extract (50–500 μg/mL), and then challenged with tert-butyl hydroperoxide (0.2 mM) for 6 h; this did not achieve ROS basal levels. The same tendency was found for the flavonoids hesperidin, hesperetin, and nobiletin (10 μg/mL) [22]. ROS generation of mandarin bioaccessible fraction of Clementine mandarin pulp has been studied by other authors using Caco-2 cells, finding a significant decrease (*p* < 0.05) in ROS formation when pre-treated with bioaccessible fraction and then challenged with H_2_O_2_ 5 mM for 2 h; this did not achieve a basal ROS level [16]. In contrast with the latter studies, the samples studied in the current work showed to achieve ROS basal levels (ROS physiological levels in the order of those found for the sample without treatment with tert-butyl hydroperoxide called as negative control in this study) under pre-treatment and pre-treatment and co-administration conditions.

During digestion, some phenolic compounds could remain unchanged and others could change their chemical structure as a consequence of pH changes [7]. Particularly, citrus phenolic acids such as ferulic acid seem to increase after digestion, and others such as vanillic and p-coumaric acids decrease after digestion [23]. Regarding citrus flavonoids, hesperidin and naringin seem to decrease after digestion [23]. Still, the remaining phenolic acids and flavonoids could be absorbed by the intestinal cells and exert their biological effects.

The results suggest that phenolic compounds, including hesperidin, which have been detected in the samples under study (Table 1) could contribute to the reduction in cellular oxidative stress observed in the present investigation. Previous studies indicate that these compounds could be converted to chalcones during the digestion process and/or their native form and metabolites could be absorbed protecting cells from oxidative stress [16,23]. Flavonoids from Clementine mandarin and Navel orange pulps, such as hesperidin, has shown to remain bioaccessible after the digestion process [16]. Therefore, this compound may be absorbed and be a contributor to the antioxidant effect found in IEC-6 and CCD-18Co cells in the present investigation (Figure 2 and Figure 3). Moreover, a study conducted on the bioaccessibility and cell uptake of phytochemicals from fresh citrus fruits showed the human intestinal HepG2 cellular uptake of flavonoids (hesperidin, naringenin, narirutin, and neohesperidin) and phenolic acids (hydroxybenzoic acids) [23]. Hesperidin, narirutin, and naringin have been shown to decrease after in vitro simulation of digestion, which is partially explained by their transformation into chalcones by alkaline conditions, and by naringenin in HepG2 cells being positively correlated with cellular antioxidant capacity under induced oxidation with AAPH [23]. Specifically, the uptake of hesperidin and narirutin from Navel orange fresh fruit was reported in HepG2 cells [23]. Thus, the results of the current work are in tune with the latter study supporting the intestinal cells uptake of flavonoids present in the bioaccessible fractions of Clemenule, Ortanique, Navel, and Valencia citrus pomaces. To the best of our knowledge, this is the first report on the antioxidant effect in normal human intestinal cells by citrus bioaccessible fractions.

### 2.3. Bioaccessibility of Anti-Inflammatory Compounds Composing Citrus Pomaces

**Figure 4:**
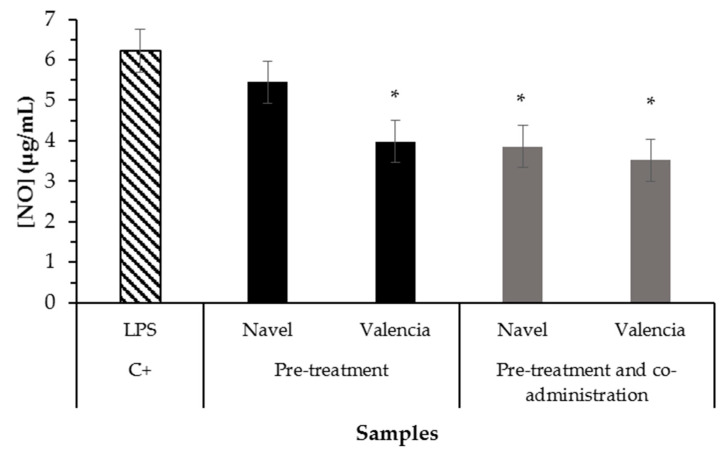
Effect of orange pomace bioaccessible fractions (250 µg/mL) on NO formation in RAW264.7 mouse macrophages. The pre-treatment assay consisted of cells treatment with samples during 24 h, followed by administration of LPS during 24 h. The pre-treatment and co-administration assay consisted of treatment with samples during 24 h, followed by co-administration of LPS and sample during 24 h. Bars represent mean values while error bars denote standard error of the mean (SEM). * Indicates statistical differences between mean values with *p* < 0.05 by Tukey test when compared to the control (C+).

The results of NO formation assay of digested raw citrus pomaces in RAW264.7 (Figure 4) showed a significant reduction (*p* < 0.05) only for orange pomaces. In the present study, the bioaccessible fraction of Valencia orange pomace showed a better anti-inflammatory capacity on LPS-induced RAW264.7 macrophages than the one from Navel orange pomace because it showed a reduction (*p* < 0.0.5) by pre-treatment and pre-treatment and co-administration assays, in contrast with Navel pomace that only showed reduction under pre-treatment and co-administration assay. This would probably be because it has a higher content of nobiletin and isosinensetin/sinensetin in the food matrix or because the higher dietary fiber content in Valencia residue may have protected anti-inflammatory compounds from digestion conditions [24].

In accordance with the current results, citrus flavonoids have been reported for their anti-inflammatory effects [25,26,27]. Hesperidin, nobiletin, and tangeretin have been shown to inhibit LPS-induced NO production collectively, as well as to possess potent anti-neuroinflammatory capacity, when studied on tangerine peel (*Citri reticulatae* pericarpium) [28]. Particularly, nobiletin (the main flavonoid present in the samples of the current work) has been reported for downregulating gene expression of pro-inflammatory cytokines (TNF-a, IL-1b, and IL-6) and iNOS suppression [28]. Hesperetin has been found to have no effect on NO production compared to control cells of macrophages isolated from the peritoneal cavity of rats without mitogen stimulation [29]. Naringenin has been found to inhibit NO production in LPS-induced RAW264.7 macrophages at a minimum concentration of 25 µg/mL, as well as attenuating ROS formation [30]. Thus, the anti-inflammatory capacity reported in this work could be associated with the orange pomace polyphenolic composition shown by the UHPLC-MS/MS results. To the best of our knowledge, this is the first time that the anti-inflammatory capacity of orange pomace after in vitro simulation of digestion has been reported through cell studies.

### 2.4. Bioaccessibility of Inhibitors of the Enzymes α-Glucosidase and α-Amylase Composing Citrus Pomaces

Results of α-glucosidase and α-amylase inhibition capacities (Table 2) confirm the presence of inhibitors of carbohydrases in raw citrus pomaces and their bioaccessible fractions, particularly α-glucosidase enzyme inhibitors. The results of the present work are in agreement with previous reports on raw citrus samples. Citrus flavonoids have shown carbohydrase inhibition capacity [31]. *Citrus × clementina* Hort. juice α-glucosidase inhibition capacity was assessed, finding an IC_50_ value of 77.79 μg/mL for the juice from hill, followed by an IC_50_ value of 93.31 μg/mL for coastal plain juice; for α-amylase inhibition capacity IC_50_, values ranged from 226.69 to 243.24 μg/mL, with no significant differences for hill and coastal plain juice, respectively [32]. Methanol and aqueous extracts of sweet orange pomace have been found to possess 10–12% and 8–11% α-amylase inhibition capacity, respectively (acarbose IC_50_ = 45 µg/mL) [33]. *Citrus medica L.* cv Diamante peel extract exhibited α-amylase and α-glucosidase inhibition, with IC_50_ values of 258.7 and 263.2 µg/mL, respectively, and hesperetin IC_50_ values of 150 and 7 µM for α-amylase and α-glucosidase, respectively (acarbose IC_50_ values of 50.0 and 35.5 µg/mL) [34].

The α-glucosidase inhibition capacity of bioaccessible fractions was unchanged or increased after digestion, suggesting the release of inhibitor compounds during the digestion process. In contrast, α-amylase inhibition capacity was decreased after the digestion process, with the exception of Clemenule mandarin pomace. To the best of our knowledge, this is the first report on inhibitors of carbohydrases from citrus pomace bioaccessible fractions.

### 2.5. Bioaccessibility of Bioactive Compounds Composing Biscuits Containing Orange Pomace as a Food Ingridient

**Figure 5:**
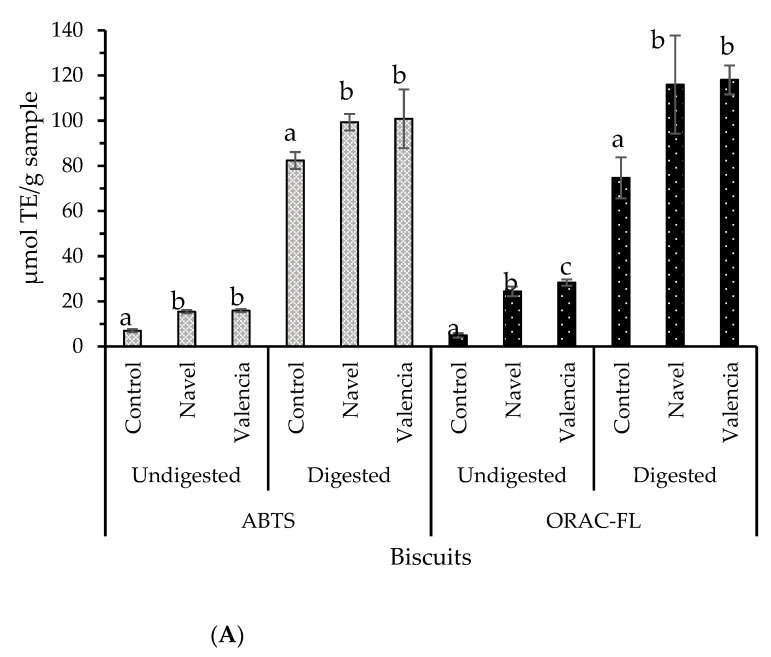

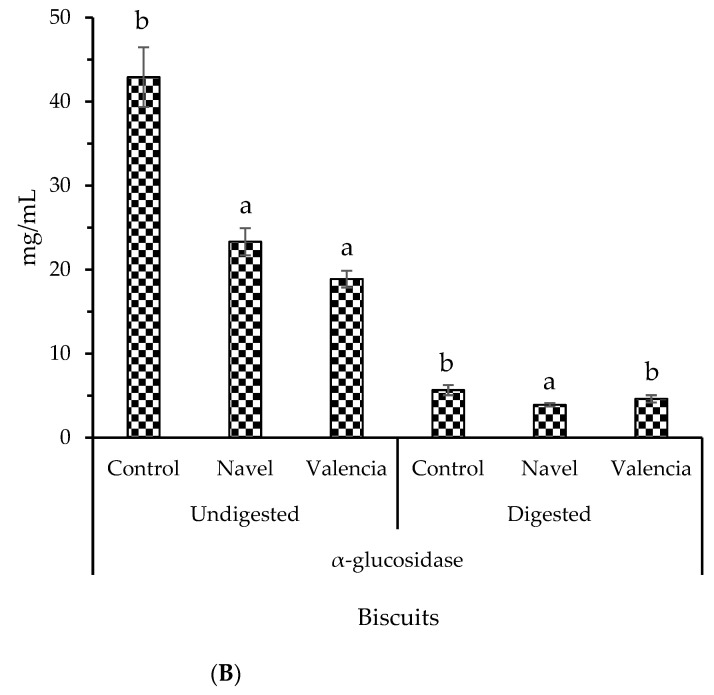
(**A**) Antioxidants and (**B**) inhibitors of α-glucosidase composing biscuits based on orange pomaces and their bioaccessible fractions. Bars represent mean values while error bars represent the standard deviation (*n* = 3). Different letters (a, b and c) indicate significant differences (Tukey, *p* < 0.05) between values in each set of six samples, where sets of samples are undigested ABTS samples, digested ABTS samples, undigested ORAC-FL samples, digested ORAC-FL samples, undigested α-glucosidase samples, and digested α-glucosidase samples.

Antioxidant compounds and inhibitors of α-glucosidase remained bioaccessible after biscuit in vitro simulated digestion containing orange pomaces as an ingredient (Figure 5). The results are in accordance with other reports of biscuits containing other by-products [35]. Biscuits showed improved antioxidant capacity (ABTS and ORAC-FL) by the addition of orange pomaces when compared to control biscuits (*p* < 0.05). Antioxidant capacity and α-glucosidase activity results seem to indicate bioactive compounds’ protection against degradation during the digestive process by means of the biscuit food matrix. Navel orange pomace displayed significantly higher (*p* < 0.0.5) α-glucosidase inhibition capacity (Figure 5), representing the best candidate as an ingredient in the formulation of biscuits to reduce the risk of diabetes.

### 2.6. Food Sensory Quality

**Figure 6:**
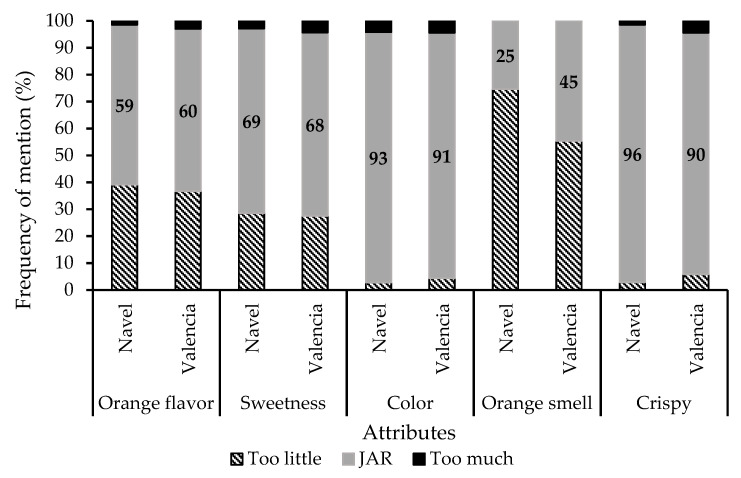
Sensory evaluation study with consumers (*n* = 75) of the biscuits with orange pomace using CATA + JAR. Frequency distribution in percentage for just-about-right scores for each dimension (too little, JAR, too much) and each attribute. The number represents the percentage of consumers who selected an attribute as “just about right”.

The overall consumer acceptance of Navel and Valencia orange pomace biscuits was 6.86 and 6.68 (scale 1–9), respectively, with no significant differences (*p* > 0.05). Valencia and Navel orange pomace biscuits presented very similar sensory profiles (Figure 6), except for the intensity of flavor, being less soft, more intense, and more persistent for Valencia. These biscuits were characterized by having little to adequate orange flavor, little to adequate sweetness, bitter, natural flavor, adequate color, little to adequate smell of orange, homemade (frequency of mention 74 and 70% for Navel and Valencia orange pomace biscuits, respectively) and adequate crispy. Both biscuit orange varieties presented a typical orange color, implying it could be applied as a natural food dye in line with consumers’ concerns about toxicity, food allergies, side effects of synthetic food dyes, and the concern for health-promoting foods consumption [36]. Orange pomace could suit all these consumer concerns.

Pearson correlations showed strong positive correlations between overall acceptance and certain attributes that comprise adequate orange flavor, adequate sweetness, natural flavor; middle positive correlations involved adequate orange smell, homemade, soft flavor, soft, fluffy; and low positive correlations comprised adequate color, too dark color, persistent flavor, pasty, moist, little crispy, adequate crispy. Strong negative correlation comprised little orange flavor, little sweetness, strange flavor; middle negative correlations comprised too much orange flavor, bitter, synthetic flavor with aftertaste, industrial, strong flavor; low negative correlations comprised too sweet, salty, too light color, little orange smell, hard, sandy, dry, shelling, too crispy. Adequate orange flavor was positively correlated with adequate sweetness, natural flavor, and adequate orange smell. Adequate sweetness was positively correlated with natural flavor and negatively with aftertaste, indicating adequate sweetness may hide aftertaste and strange flavor. Bitter taste was positively correlated with aftertaste, indicating orange pomace bitter compounds are responsible for the aftertaste and the soft flavor, as well as positively correlated with a strange flavor. Synthetic flavor was positively correlated with the attributes of industrial and strange flavor, possibly because of the sucralose influence on the flavor. Aftertaste was positively correlated with strange flavor. The attribute industrial was negatively correlated with natural flavor and positively correlated with aftertaste, strange flavor, and pastiness, meaning the latter attributes resulted to represent the consumers’ perception about the attribute industrial. The feature hard was positively correlated with dryness.

Other authors have studied the sensory profile of cookies with mango peel powder, resulting in lower acceptability when augmenting its addition, as well as the score of the tested attributes (crust color, crust appearance, crumb color, texture, taste/flavor, and overall quality) with the subsequent bitter taste due to high polyphenol content [37]. Additionally, mango peel powder was incorporated (5%) into semolina-yielded macaroni, enhancing the nutritional quality of macaroni without compromising its textural and sensory properties [38]. In addition, orange pomace has also been incorporated into macaroni, finding diminished overall acceptability along with taste and aftertaste, possibly because of the bitterness of the orange by-product, but still presenting market potential when fresh pasta was supplemented with 25 g/kg and 50 g/kg of orange fiber [39]. The latter results are in agreement with the results of this study.

Altogether, the results support the sensory quality of the formulated orange pomace biscuits and their potential to reduce the risk of diabetes. The achieved new biscuit formulation is sugar-free and contains dietary fiber (from orange pomace and inulin), as well as bioactive compounds that may have the potential to reduce the risk of diabetes by remaining bioaccessible after food digestion.

## 3. Materials and Methods

### 3.1. Materials

All the reagents used were of reagent grade. Reagents for antioxidant capacity were purchased from Sigma-Aldrich (St. Louis, MO, USA): 2,20-azinobis-(3-ethylbenzothiazoline-6-sulfonic acid) diammonium salt (ABTS), 6-hydroxy-2,5,7,8-tetramethylch-roman-2-acid (Trolox), fluorescein (FL) disodium salt, and 2,20-azobis (2-methylpropionamidine) dihydrochloride (AAPH). Reagents for carbohydrases enzymatic activity were purchased from Sigma-Aldrich (St. Louis, MO, USA): α-glucosidase from rat intestine acetone powder, acarbose, 4-methylumbelliferyl-α-d-glucopyranoside, α-amylase from human saliva (type IX-A), starch, maltose standard, and 3,5-dinitrosalicylic acid. Digestion enzymes were purchased from Sigma-Aldrich (St. Louis, MO, USA).

RAW264.7 mouse macrophage cells and the normal human colon fibroblast cell line (CCD-18Co) were obtained from American Type Culture Collection (ATCC, Manassas, VA, USA). IEC-6 rat small intestine epithelial cell line were kindly provided by the Bioanalytical Techniques Unit (BAT) of the Instituto de Investigación en Ciencias de la Alimentación (CIAL) (Madrid, Spain). Dulbecco’s modified Eagle medium (DMEM), heat inactivated fetal bovine serum (FBS) (10% *v*/*v*), L-Glutamine (1% *v*/*v*), and antibiotics (penicillin and streptomycin 1:1, 1% *v*/*v*) were used for cultivating the cells, and were purchased from Gibco Laboratory (Invitrogen Co, Grand Island, NY, USA), except for FBS, which was purchased from Hyclone (USA). 3-(4,5-dimethylthiazol-2-yl)-2,5-diphenyltetrazolium bromine (MTT) was purchased from Sigma-Aldrich (St. Louis, MO, USA). For ROS level determination, the oxidant-sensitive probe 2′.7′-dichlorofluorescin diacetate (DCFH-DA) was obtained from Sigma-Aldrich (St. Louis, MO, USA). For nitric oxide measurements, sulfanilamide, *N*-(1-napthyl)ethylenediamine dihydrochloride, phosphoric acid, sodium nitrite, and lipopolysaccharide from E. coli O55:B5 (LPS) were purchased from Sigma-Aldrich (St. Louis, MO, USA).

### 3.2. Samples

Raw material: Citrus pomaces from Clemenule and Ortanique mandarin varieties and Navel and Valencia orange varieties were provided by Azucitrus (Paysandú, Uruguay). Mandarin and orange pomaces, composed of a wet mixture of pulp, peels, and seeds, were freeze-dried for 4 days to a constant weight (under 8% of moisture), milled, and stored in a freezer at −20 °C for further analysis in closed plastic bags with moisture indicator control. Chemical characterization of raw materials is shown in Table S1.

Food: Biscuits containing citrus pomaces as food ingredients (10% *w/w* on wet dough basis) were prepared according to the formulation shown in Table 3. The mixture was baked at 180 °C for 12 min. The biscuits were milled and stored in a freezer at −20 °C for further analysis.

### 3.3. Methods

**Figure 7:**
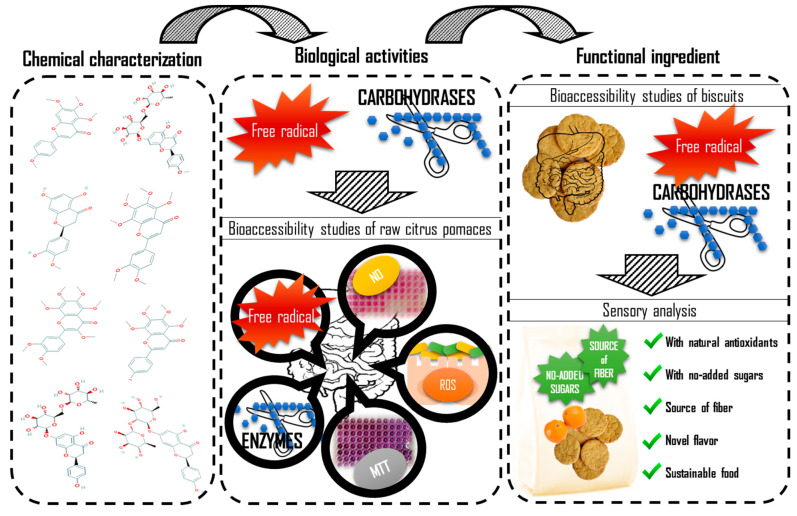
Scheme of the steps followed in the current work.

Figure 7 shows the steps followed in the present work to better understand the methods carried out.

#### 3.3.1. Analysis of Individual Phenolic Compounds Composing Citrus Pomaces

Solutions of 10 mg/mL in H_2_O:MeOH (50:50, *v/v*) of Clemenule and Ortanique mandarin and Navel and Valencia orange pomaces were analyzed by UHPLC-MS/MS. The analysis of phenolic compounds was carried out according to the method described by Fernández-Fernández et al. [19]. Briefly, the identification and quantification of these compounds was performed using a Thermo Ultimate™ 3000 HPLC (Thermo Scientific, Sunnyvale, CA, USA) coupled to a hybrid quadrupole-orbitrap mass spectrometer (Q-ExactiveTM; Thermo Scientific, Bremen, Germany), equipped with heated electrospray ionization (HESI-II). Chromatographic separation was carried out using a UPLC BEH C18 column; 2.1 mm × 100 mm, 1.7 μm particle size (Waters, Milford, MA, USA) using water-acetonitrile gradient according to Barnaba et al. [40] at a flow rate of 0.3 mL/min. Data acquisition and processing were carried out with Thermo Scientific™ Dionex™ Chromeleon™ 7.2 Chromatography Data System (CDS) software. Qualitative and semi-quantitative analyses of the mass spectrometry data were performed using full SCAN approach. For the screening analysis, the accurate mass of both precursor and product ions and their isotope patterns were used. In absence of standards, the peak area was used as the parameter to define the relative concentration.

#### 3.3.2. Analysis of Bioaccessibility of Health Promoting Compounds

Prior to the analysis of the samples, citrus pomaces and biscuits were in vitro digested, mimicking the human oral gastrointestinal conditions as described by Hollebeeck et al. [41]. Bile salts were removed by using cholestyramine resin [19]. The desalted samples were frozen and stored for further analysis.

##### Antioxidant Compounds

The effect of the digestion process on the bioaccessibility of antioxidants was determined by analysis of the Total Phenol Content (TPC) by Folin reaction and the overall antioxidant capacity of the samples was performed by employing ABTS and ORAC-FL assays as described by Fernández-Fernández et al. [42].

Folin–Ciocalteau method was performed as described by Slinkard and Singleton [43]. Gallic acid standard curve (0.05 to 1.0 mg/mL) was built. Samples were prepared according to Fernández-Fernández et al. [42]. Briefly, for better polyphenols extraction, 120 µL of DMSO and 1880 µL of distilled water (2 mL end volume) were added to samples. Results were expressed as mg GAE/g digest and all determinations were performed in triplicate.

ABTS and ORAC-FL methods were carried out as described by Fernández-Fernández et al. [42]. Solutions of samples were prepared in a 2 mL eppendorf tube in distilled water, followed by vortex for 2 min and centrifuged at 9500 rpm for 10 min (room temperature). Supernatants samples were added to flat-bottom 96-well plates, for ABTS translucent and ORAC-FL black plates. ABTS method was performed by adding 10 µL of supernatants and 190 µL of ABTS working solution, followed by measuring absorbance at 750 nm in a Thermo Scientific FC microplate reader. ORAC-FL method was performed by measuring fluorescence at λ_excitation_ = 485 nm and λ_emission_ = 520 nm in a Varioskan Lux (Thermo Scientific) fluorimeter microplate reader. Trolox calibration curves were constructed for both methods, ranging from 0.25 to 1.5 mM and 0.1 to 0.8 mM for ABTS and ORAC-FL, respectively. Results were expressed as μmol TE/g digest. Assays were performed at least in triplicate.

Reactive Oxygen Species (ROS) formation on IEC-6, CCD-18Co and RAW264.7 cells was performed for digested raw citrus pomaces, as described by Fernández-Fernández et al. [19], to obtain further information on the antioxidant properties of those compounds of the citrus byproduct remaining bioaccessible after its digestion. Briefly, sterile 96-well plates were seeded and incubated for 24 h with 80,000 RAW264.7, 20,000 IEC-6, and 10,000 CCD-18Co cells/well. Then, different concentrations of the samples after in vitro simulation of digestion were added to each well (150 µL) for 24 h. Induced formation of ROS was achieved by tert-butyl hydroperoxide 1 mM (oxidant agent). Afterwards, 2 µL of DCFH-DA probe (5 mg/mL in DMSO) were added to each well and incubated for 30 min, followed by supernatant removal, one cell wash with PBS, and the same concentrations (150 µL) were added to each well. Fluorescence was measured in a fluorimeter microplate reader (λ_excitation_ = 485 nm, λ_emission_ = 528 nm). After measuring fluorescence, cell viability (MTT assay) was performed in the same plate by adding 20 µL of MTT (6 mM) reagent (as described below) to correct ROS values by using the following Equation (1) with the raw values of absorbance (MTT assay) and fluorescence (ROS assay):

(1) $$\%\;ROS=\frac{Fluorescence_{sample}}{Absorbance_{MTT\;sample}}*\frac{Absorbance_{MTT\;C-}}{Fluorescence_{C-}}*100$$

Before performing cell studies, cell viability was evaluated by MTT assay, as described by Fernández-Fernández et al. [42], in order to determine the concentration range of the samples, obtaining more than 80% cell viability. Cells were seeded (quantity of cells stated above) in sterile 96-well plates and incubated in the same conditions for 24 h. Then, cells were incubated with different concentrations of the samples for 24 h (150 µL), followed by the addition of 20 µL of MTT (6 mM) reagent and different incubation times, depending on cell type: RAW264.7 cells (30 min), IEC-6 cells (3 h), and CCD-18Co cells (3 h). Afterwards, supernatants were removed, 100 µL of DMSO was added to each well, and plates were incubated for 5 min to achieve better homogenization. Absorbance was measured in a microplate reader at 570 nm and viability percentage was calculated by considering control absorbance (non-treated cells, DMEM) as 100%.

##### Anti-Inflammatory Compounds

The anti-inflammatory potential of the bioaccessible compounds of raw citrus pomaces was determined by measuring nitric oxide (NO) production induced by lipopolysaccharide (LPS) in RAW264.7 mouse macrophages, as described by Fernández-Fernández et al. [42]. Briefly, different concentrations of samples (150 µL) were placed on each well, followed by incubation of 24 h and LPS stimuli for another 24 h (pre-treatment assay). Alternatively, incubation with different concentrations of samples for 24 h, followed by LPS+sample incubation for another 24 h (pre-treatment and co-administration assay), was also performed. Afterwards, a 15 min reaction of 100 µL of cell supernatants and 100 µL of Griess reagent at room temperature was performed for measuring NO production by absorbance measurement at 550 nm. Sodium nitrite standard curve was built in a range of 0 to 10 µg/mL. Negative (DMEM) and positive (DMEM+LPS) controls were also tested.

##### Inhibitors of Carbohydrases Enzymatic Activity

The bioaccessibility of compounds able to modulate the metabolism of carbohydrates, also called antidiabetic compounds, was estimated by measuring the enzymatic activity of α-glucosidase and α-amylase, as described by Fernández-Fernández et al. [19], to evaluate antidiabetic activity using a Varioskan Lux (Thermo Scientific) fluorimeter microplate reader. Then, α-glucosidase inhibition capacity was achieved by measuring fluorescence of 4-MUF-α-D-glucopyranoside probe in black 96-well plates at 37 °C each minute for 30 min at 360 ± 40 nm and 460 ± 40 nm of excitation and emission wave lengths, respectively. Human α-amylase (35 U/mL, Sigma powder 160 U/mg, 0.44 mg in 2 mL, final activity 2.5 U/mL) inhibition capacity was performed by measuring starch hydrolysis with human α-amylase, 1% *w/v* starch stock solution, and dinitrosalicylic acid color reagent in 96-well plates at 540 nm in a microplate reader (200 μL of volume per well) Varioskan Lux. Samples were prepared in 20 mM sodium phosphate buffer at pH 6.9. The reaction was performed in 1.5 mL eppendorf tubes in a water bath at 37 °C. Sample and enzyme blanks were measured, as well as negative and positive controls. Acarbose was used as the pharmaceutical of reference (probed inhibition capacity) for both assays. Dose–response curves were constructed (% inhibition vs. [sample or standard] (mg/mL)) in order to obtain IC_50_ value. Inhibition percentages were calculated as shown in the following equation:

(2) $$\%\;\alpha -glucosidase\;Inhibition=\frac{F_{NC}-F_{s/a}}{F_{NC}}\times 100$$

(3) $$\%\;\alpha -amylase\;Inhibition=\frac{A_{C+}-\left(A_{s/a}-A_{b}\right)}{A_{C+}}\times 100$$

where *F_NC_* is the fluorescent measurement of negative control and *F_s/a_* is the fluorescent measurement of sample/acarbose minus sample/acarbose blank; *A_C+_* is the absorbance measurement of positive control; *A_s/a_* is the absorbance of sample/acarbose, enzyme, starch, and dinitrosalicylic acid; and *A_b_* is the absorbance of sample with buffer and dinitrosalicylic acid (blank).

#### 3.3.3. Assessment of Food Sensory Quality

Food quality of the novel biscuits was evaluated by sensory analyses employing 75 consumers (40% male and 60% female, aged between 18 and 87 years old) recruited at Departamento de Ciencia y Tecnología de Alimentos (Facultad de Química, UdelaR, Uruguay). The two samples evaluated were the biscuits with the addition of 10% *w/w* of Navel and Valencia pomace dry powder. Analysis was performed through a CATA (check all that apply) + JAR (just about right) and overall acceptance. The attributes evaluated included “overall liking”, “taste”, “visual appearance”, “texture”, and “smell”. Different biscuit formulations were evaluated according to the frequency of the mentions of the different attributes analyzed. Overall acceptance was evaluated on a scale of 1 to 9. Consumers were informed about the composition of the biscuit with the novel ingredient (citrus pomace) by the following phrase: “free of sugar biscuits containing antioxidants and dietary fiber from oranges”. Biscuits were served in black plastic cups.

#### 3.3.4. Statistical Analysis

Statistical differences between the mean values of the studied parameters were determined by the Tukey test (*p* < 0.05) using Infostat v. 2015 program and represented by different letters. In cell studies, the statistical differences between mean values of samples when compared to the control with *p* < 0.05 were indicated with an asterisk. Results were expressed as mean ± standard deviation (SD) (*n* = 3). Sensory statistical analysis was performed using the program XLStat-Sensory v. 2017.

## 4. Conclusions

Nobiletin was the main flavonoid present in raw mandarin and orange pomaces determined by UHPLC-MS/MS analysis, followed by other flavonoids such as hesperidin/neohesperidin, tangeretin, heptamethoxyflavone, tetramethylscutellarein, and naringin/narirutin. In vitro simulation of digestion showed the bioaccessibility of antioxidants composing raw citrus pomaces by TPC, ABTS, and ORAC-FL results, as well as inhibitors of carbohydrases, with a marked effect on the α-glucosidase enzyme. Mandarin pomace bioaccessible compounds significantly inhibited (*p* < 0.05) induced intracellular ROS formation in normal rat small intestinal cells (IEC-6 cells). In the case of orange pomace bioaccessible compounds, a significant inhibition (*p* < 0.05) of the induced intracellular ROS formation in normal human intestinal cells (CCD-18Co cells) was observed, as was reduced (*p* < 0.05) nitric oxide production on LPS-induced RAW264.7 macrophages, indicating its potential as an anti-inflammatory agent. A new formulation of sugar-free biscuits containing dietary fiber and bioactive compounds (antioxidant and inhibitors of α-glucosidase) from raw orange pomaces showed potential for a reduction in diabetes risk. Sensory analysis showed raw orange pomaces as suitable ingredients for biscuits, since they were well accepted by consumers. In conclusion, the in vitro bioactivity displayed by the bioaccessible fractions of raw citrus pomaces under study may be associated with their flavonoid composition. Particularly, our results support the potential of unfractionated orange pomaces as functional ingredients for diabetes risk reduction.

## Figures and Tables

**Table 1:** Data on identification of phenolic compounds composing citrus pomaces.

Compounds ^1^	Clemenule Mandarin ^2^	Ortanique Mandarin ^2^	Navel Orange ^2^	Valencia Orange ^2^	RT	λmax (nm) (UHPLC-DAD)	[M + H]^+^ (m/z)	Fragments (m/z)
nariturin-4-glucoside/naringin glucoside	0.000078	0.000391	0.000755	0.000528	9.6	266sh, 359	741.2248	271.0639, 151.0035
Rutin	0.003400	0.005722	0.001625	0.001366	10.3	285, 325	609.1461	301.0350, 271.0257
Eriocitrin/ Neoeriocitrin 1	0.001715	0.000608	0.001164	0.000806	10.6	266, 336/268, 334	595.1668	287.0580, 151.0034
Rhoifolin/Isorhoifolin	0.000855	0.000944	0.000236	0.000086	11.0	284, 330/284	579.1708	271,0595
Naringin/Narirutin	0.008453	0.029204	0.030457	0.021736	11.2	250, 268, 342	579.1719	271.0637, 151.0035
Diosmin isomer 1	0.000783	0.001677	0.000839	0.000797	11.3	285, 340/284	607.1668	299.0580, 284.0338
Eriocitrin/ Neoeriocitrin 2	0.000187	0.000048	0.000060	0.000056	11.3	268, 342	595.1668	287.0580, 151.0034
Diosmin isomer 2	0.000889	0.000327	0.000441	0.000393	11.4	283, 326/283, 332	607.1668	299.0580, 284.0338
Hesperidin/ Neohesperidin	0.059179	0.046458	0.071641	0.064532	11.6	283, 328/266sh, 354	609.1825	301.0739, 151.0035
Poncirin/ Isosakuranetin-7-*O*-rutinoside	0.000270	0.002624	0.002497	0.001776	13.1	270sh, 338/214, 328/285, 331	593.1876	285.0763
Isosinensetin/ Sinensetin/ Tangeretin 1	0.015972	0.015961	0.010084	0.010829	16.2	270sh, 338/214, 328/285, 331	373.1282	343.0806, 153.0181
Isosinensetin/ Sinensetin/ Tangeretin 2	n.d.	0.048355	0.058543	0.065486	16.7	272, 324	373.1282	343.0806, 153.0181
Nobiletin	0.081665	0.099307	0.073267	0.079792	17.3	268sh, 342	403.1387	373.091, 183.0288
Heptamethoxyflavone	0.072137	0.010010	0.019473	0.018539	17.6	272, 302	433.1493	403.1019, 418.1251
Tetramethylscutellarein	0.033105	0.085489	0.049083	0.047840	17.7	270sh, 338/214, 328/285, 331	343.1176	313.0701, 153.0180
Isosinensetin/ Sinensetin/ Tangeretin 3	0.035461	0.072075	0.024060	0.024608	18.1	266sh, 359	373.1282	343.0806, 153.0181
TIC	823166557	884024903	878913863	817363291				

n.d.: not detected. sh: shoulder. ^1^ Compound Discoverer 3.1 (mzCloud library, Advanced Mass Spectral Database). ^2^ Results normalized with TIC area (area/area TIC).

**Table 2:** Inhibitors of the enzymes α-glucosidase and α-amylase from raw citrus pomaces and bioaccessible fractions.

Samples	α-Glucosidase (IC_50_, mg/mL)		α-Amylase (IC_50_, mg/mL)	
	Undigested	Digested	Undigested	Digested
Clemenule mandarin	4.92 ± 0.27 ^b^	3.97 ± 0.97 ^a^	70.19 ± 11.16 ^bc^	58.04 ± 2.09 ^a^
Ortanique mandarin	3.42 ± 0.64 ^a^	4.93 ± 0.41 ^a^	50.07 ± 2.42 ^ab^	105.68 ± 16.03 ^b^
Navel orange	10.84 ± 1.19 ^c^	11.42 ± 0.89 ^b^	5.19 ± 0.22 ^a^	62.00 ± 1.62 ^a^
Valencia orange	5.19 ± 1.98 ^b^	5.09 ± 0.39 ^a^	77.57 ± 15.27 ^c^	101.17 ± 4.70 ^b^

Results are expressed as mean values ± SD (*n* = 4). Different letters (a, b and c) indicate significant differences (Tukey, *p* < 0.05) between values in the same column in each set of four samples where sets of samples are undigested α-glucosidase samples, digested α-glucosidase samples, undigested α-amylase samples, and digested α-amylase samples. IC_50_, half maximal inhibitory concentration.

**Table 3:** Biscuit formulations.

Ingredients	Control	Navel	Valencia
	g/100 g		
Butter	10	10	10
Sunflower oil	4.25	4.25	4.25
Egg	14	14	14
Baking powder	0.5	0.5	0.5
Salt	0.08	0.08	0.08
Sweetener	4	4	4
Wheat flour	62.17	52.17	52.17
By-product	0	10	10
Inulin	5	5	5
Total	100	100	100

## Data Availability

We do not have supplementary data to show other than the results presented in the “Results and Discussion” section.

## Supplementary Materials

The following are available online, Figure S1: Representative extracted ion chromatograms (EICs) for both positive and negative electrospray ionization, Table S1: Proximate analysis of raw citrus pomaces powders after drying and milling.

## Sample Availability

Samples of the compounds are available from the authors.
