# Supplementary material for: In Vitro Bioaccessibility of Bioactive Compounds from Citrus Pomaces and Orange Pomace Biscuits
Source: Molecules. 2021 Jun 8;26(12):3480. doi: 10.3390/molecules26123480 (PMC8229244; doi:10.3390/molecules26123480)
Supplement: Supplementary file 1 [file molecules-26-03480-s001.zip › molecules-1218049-supplementary.pdf]

molecules

MDPI

Supplementary material

Negative MS quantitation channels

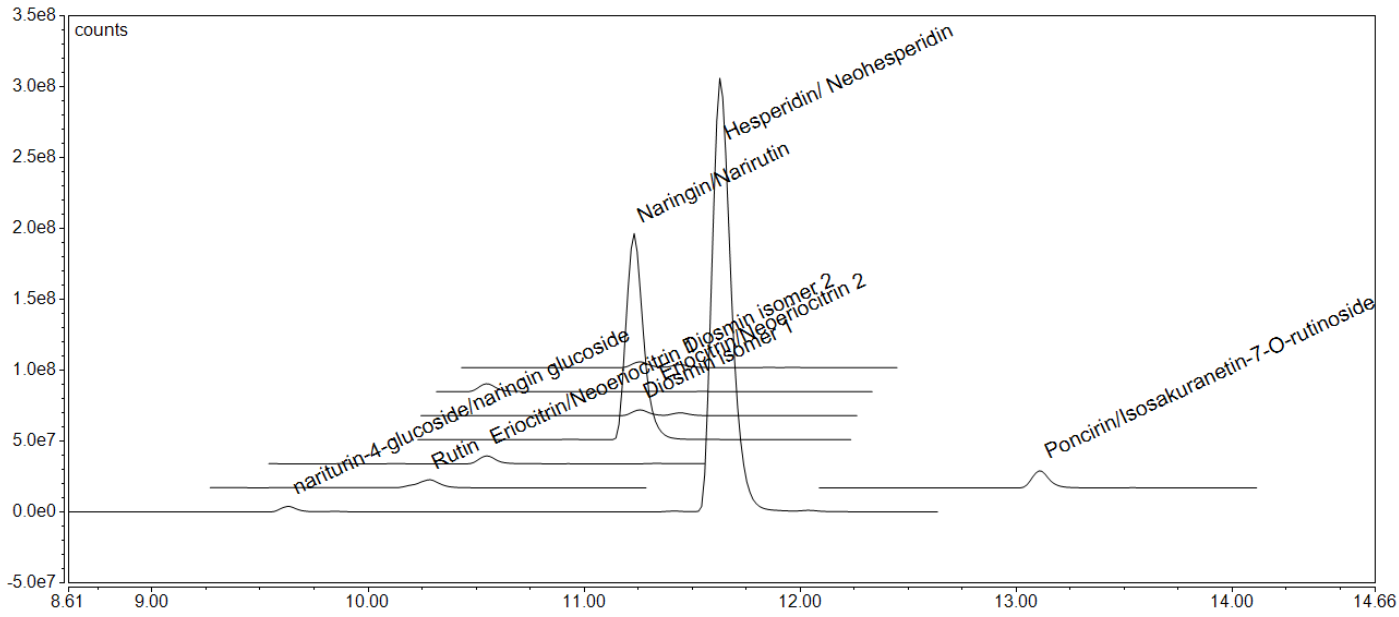

Positive MS quantitation channels

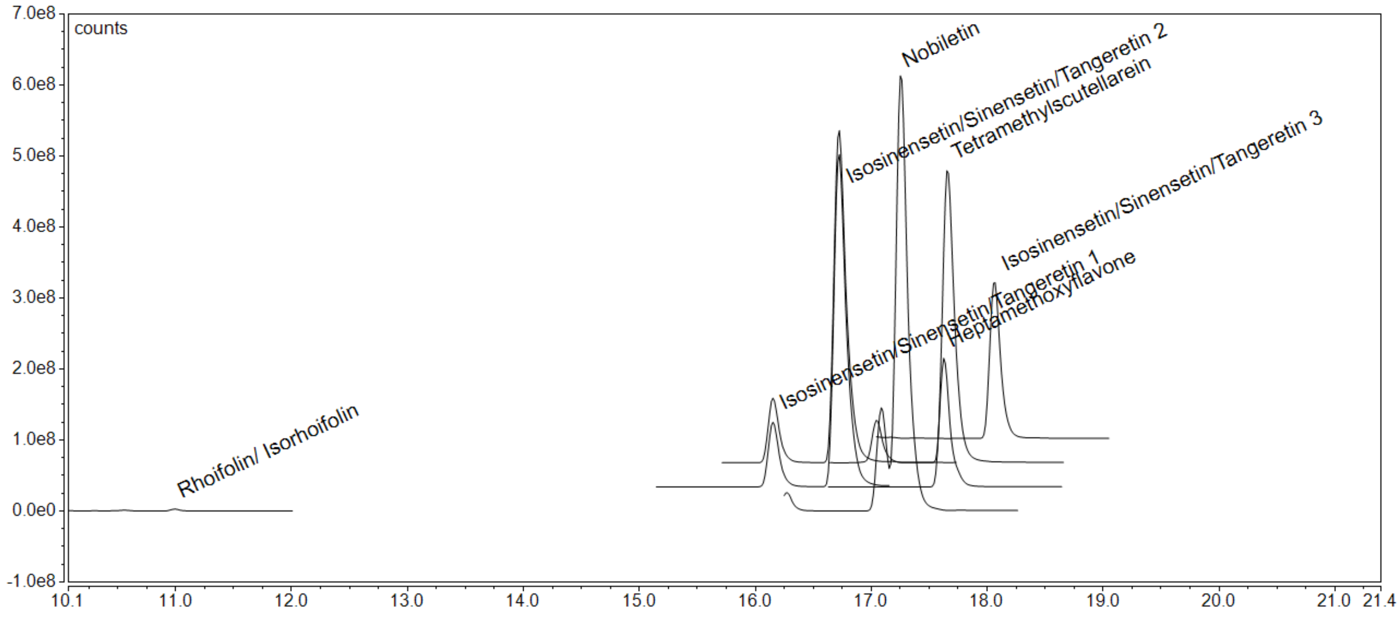

Figure S1. Representative extracted ion chromatograms (EICs) for both positive and negative electrospray ionization

Table S1. Proximate analysis of raw citrus pomaces powders after drying and milling.

Components	Clemenule Mandarin	Ortanique Mandarin	Navel Orange	Valencia Orange
Lipids	0.79	1.41	0.85	1.18
Carbohydrates	84.77	80.73	83.16	83.62
Sugars	41.06	42.14	47.96	31.91
Fiber	43.71	38.59	35.20	51.71
Proteins	5.63	6.64	5.09	4.89
Ashes	3.10	3.22	2.69	2.99

Results are expressed as mean values as g/100 g of dry pomace powder.
